# Thioredoxin Glutathione Reductase from *Schistosoma mansoni:* An Essential Parasite Enzyme and a Key Drug Target

**DOI:** 10.1371/journal.pmed.0040206

**Published:** 2007-06-19

**Authors:** Angela N Kuntz, Elisabeth Davioud-Charvet, Ahmed A Sayed, Lindsay L Califf, Jean Dessolin, Elias S. J Arnér, David L Williams

**Affiliations:** 1 Department of Biological Sciences, Illinois State University, Normal, Illinois, United States of America; 2 Biochemie-Zentrum der Universität Heidelberg, Heidelberg, Germany; Queensland Institute of Medical Research, Australia; 3 Centre National de la Recherche Scientifique (CNRS), Paris, France; 4 Institut Européen de Chimie et Biologie, CNRS UMR 5144, Bordeaux University, Pessac Cedex, France; 5 Medical Nobel Institute for Biochemistry, Department of Medical Biochemistry and Biophysics, Karolinska Institutet, Stockholm, Sweden

## Abstract

**Background:**

Schistosomiasis—infection with helminth parasites in the genus *Schistosoma,* including S. mansoni—is a widespread, devastating tropical disease affecting more than 200 million people. No vaccine is available, and praziquantel, the only drug extensively utilized, is currently administered more than 100 million people yearly. Because praziquantel resistance may develop it is essential to identify novel drug targets. Our goal was to investigate the potential of a unique, selenium-containing parasite enzyme thioredoxin glutathione reductase (TGR) as a drug target.

**Methods and Findings:**

Using RNA interference we found that TGR is essential for parasite survival; after silencing of TGR expression, in vitro parasites died within 4 d. We also found that auranofin is an efficient inhibitor of pure TGR (*K*_i_ = 10 nM), able to kill parasites rapidly in culture at physiological concentrations (5 μM), and able to partially cure infected mice (worm burden reductions of ~60%). Furthermore, two previously used antischistosomal compounds inhibited TGR activity, suggesting that TGR is a key target during therapy with those compounds.

**Conclusions:**

Collectively, our results indicate that parasite TGR meets all the major criteria to be a key target for antischistosomal chemotherapy. To our knowledge this is the first validation of a *Schistosoma* drug target using a convergence of both genetic and biochemical approaches.

## Abstract 2

Using both genetic and biochemical approaches, David Williams and colleagues show that the parasite thioredoxin glutathione reductase meets all the major criteria to be a key target for antischistosomal chemotherapy.

## Editors' Summary

**Background.:**

More than 200 million people are infected with schistosomes, a type of parasitic worm. Schistosomes have a complex life cycle that starts with them reproducing in freshwater snails. The snails release free-swimming, infectious parasites that burrow into the skin of people who swim in the contaminated water. Once in the human host, the parasites turn into larvae and migrate to the liver where they become juvenile worms. These mature into 10- to 20-mm-long adult worms and take up long-term residence in the veins draining the gut *(Schistosoma mansoni* and *S. japonicum)* or bladder *(S*. *haematobium)*. Here, the worms mate and release eggs, some of which pass into the feces and so back into water where they hatch and infect fresh snails. Schistosomiasis causes serious health problems (including chronic liver, gut, bladder, and spleen damage) in about 20 million people, making it a disease of great public-health and socioeconomic importance in the developing countries in which it mainly occurs.

**Why Was This Study Done?:**

The only drug available to treat schistosomiasis is praziquantel. Although it is very effective, people regularly get reinfected and need to be retreated once or twice a year. All told, 100 million people are currently being treated with praziquantel. Reliance on a single drug, however, is problematic, as the parasites are likely to develop resistance to the drug over time. The identification of new drug targets in schistosomes is therefore an urgent goal. In this study, the researchers have investigated whether thioredoxin glutathione reductase (TGR), a parasitic enzyme with several functions, might be a key target for antischistosomal chemotherapy. They chose this enzyme because adult worms need to make antioxidants (chemicals that prevent oxygen from damaging cells) to protect themselves against the human immune response. Antioxidant production in these worms depends on TGR; in mammalian cells, two specialized enzymes do its job. The researchers reasoned, therefore, that TGR might be an essential parasite protein and a potentially important drug target.

**What Did the Researchers Do and Find?:**

The researchers made large quantities of pure TGR and tested its activity against various substrates. The enzymatic properties and substrate preferences of TGR, they found, differed somewhat from those of its mammalian counterparts. They then screened different types of compounds for their ability to inhibit TGR. Praziquantel had no effect on TGR activity, but two antischistosomal compounds that are no longer used, potassium antimonyl tartrate and oltipraz, inhibited the enzyme. The most potent inhibitor of TGR, however, was a gold-containing complex called auranofin, low levels of which inhibited TGR in test tubes, completely killed larval, juvenile, and adult parasites living in laboratory dishes within hours, and more than halved the worm burden in infected mice. Finally, the researchers used a technique called RNA silencing to test the importance of TGR for worm survival. Fragments of double-stranded RNA (dsRNA) stop proteins being made from messenger RNA that contains an identical sequence. The addition of TGR dsRNA to larval parasites in a dish greatly reduced TGR enzyme activity and killed nearly all the parasites within days.

**What Do These Findings Mean?:**

These findings suggest TGR as a key target for antischistosomal drug development. Indeed, the discovery that two previously used antischistosomal compounds inhibit TGR suggests that the enzyme has already served as a target protein. The RNA silencing experiment shows that TGR is essential for parasite survival, and the biochemical analyses indicate that TGR and its mammalian counterparts have different substrate specificities. Thus, it should be possible to find compounds that inhibit TGR but have much less effect on the mammalian enzymes. This is certainly true for auranofin, a drug used to treat rheumatoid arthritis. Whether auranofin will be an effective treatment for schistosomiasis remains to be seen—an agent that completely kills schistosomes in animals would be preferable. However, even a 50% reduction in worm burden would decrease the human health problems caused by schistosomiasis, and a combination of auranofin (or another TGR inhibitor) with an agent that works by a different mechanism might be more effective and would also reduce the chances of the parasite developing drug resistance.

**Additional Information.:**

Please access these Web sites via the online version of this summary at http://dx.doi.org/10.1371/journal.pmed.0040206.

- World Health Organization provides information on schistosomiasis, including a fact sheet in English, Spanish, French, Arabic, Chinese, and Russian
- US Centers for Disease Control and Prevention provide information for the public and for professionals on schistosomiasis
- MedlinePlus encyclopedia includes an entry on schistosomiasis (in English and Spanish)
- The Schistosomiasis Control Initiative has information on the disease and its control
- Wikipedia has a page on schistosomiasis that is available in several languages (note: Wikipedia is a free online encyclopedia that anyone can edit)

## Introduction

Schistosomiasis (also known as bilharzia)—infection with the helminth parasites in the genus *Schistosoma*—remains an important infection in many tropical areas, especially Africa. More than 200 million people have schistosomiasis, with 20 million exhibiting severe symptoms. Recent analyses suggest that the morbidity due to schistosomiasis is grossly underestimated [1], resulting in an estimated 280,000 deaths annually in sub-Saharan Africa alone [2]. Since the mid-1980s praziquantel (Figure S1) has been the drug of choice for schistosomiasis; effectively it is currently the only choice available. Artemether has shown promise as a new drug for schistosomiasis, targeting larval parasites more effectively than praziquantel, which is primarily effective against adult parasites [3]. However, the use of artemether for schistosomiasis should be restricted so that its use as an antimalarial compound is not put at risk from the development of possible drug resistance in the malaria parasite. With the exception of the artemisinin-based drugs, no new drugs have been introduced for schistosomiasis after praziquantel, and prior drugs have ceased to be produced or are ineffective [4]. Furthermore, resistance to oxamniquine, which is effective against *S. mansoni,* has been reported [5], thereby reducing its potential usefulness. Research for new antischistosome drugs is limited by the difficulty of working with the parasite and the low priority the pharmaceutical industry generally places on tropical diseases. Currently more than 100 million people are being treated for schistosomiasis with praziquantel [2]; they are rapidly reinfected and must be retreated on an annual or semiannual basis. If praziquantel-resistant parasites develop, treatment for schistosomiasis will be in a crisis state.

**Figure 1:**
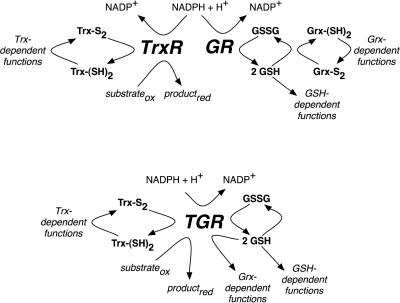
Redox Pathways in Mammals and S. mansoni In mammals (upper pathway), electrons from NADPH are transferred to an oxidoreductase flavoenzyme, either thioredoxin reductase (TrxR) or glutathione reductase (GR). Electrons are then transferred from the oxidoreductase flavoenzyme to the appropriate electron carrier, either oxidized thioredoxin (Trx-S_2_) or glutathione disulfide (GSSG) converting them to reduced thioredoxin (Trx-[SH]_2_) or glutathione (GSH), respectively. Trx-(SH)_2_ and GSH then supply reducing equivalents for a number of different reactions, including those that are glutaredoxin (Grx)-dependent. In S. mansoni (lower pathway), TrxR and GR are replaced with a unique oxidoreductase flavoenzyme, TGR, which provides reducing equivalents for Trx-, GSH- and Grx-dependent reactions.

Adult S. mansoni parasites reside in the mesenteric veins of their human hosts, where they can survive for up to 30 years [6]. Living in an aerobic environment, worms must have effective mechanisms to maintain cellular redox balance. Additionally, worms must be able to evade reactive oxygen species generated by the host's immune response. In most eukaryotes there are two major systems to detoxify reactive oxygen species, one based on the tripeptide glutathione (GSH) and the other based on the 12 kDa protein thioredoxin (Trx). In both systems reducing equivalents are provided by NADPH via dedicated oxidoreductase flavoenzymes. Glutathione reductase (GR) reduces glutathione disulfide (GSSG) and drives the GSH-dependent systems [7,8], whereas Trx reductases (TrxR) are pivotal in the Trx-dependent system (Figure 1) [9]. In addition to providing protection against oxidative damage, the Trx and GSH systems also play important roles in cell proliferation, redox regulation of gene expression, xenobiotic metabolism, and several other metabolic functions [8,9]. Because of the diverse functions of the TrxR- and GR-dependent pathways, the two oxidoreductases have been identified as promising targets for drug development for many diseases, including malaria, trypanosomiasis, and cancer [9,10].

It was recently discovered that in *S. mansoni,* specialized TrxR and GR enzymes are absent, and instead replaced by a unique multifunctional enzyme, thioredoxin glutathione reductase (TGR) (Figure 1) [11]. This reliance on a single enzyme for both GSSG and Trx reduction suggests that the parasite's redox systems are subject to a bottleneck dependence on TGR. The amino acid sequence and domain structure of schistosome TGR has similarities to mammalian forms of TrxR and GR, with an additional amino-terminal extension of a glutaredoxin (Grx) domain of ~110 amino acids with a typical CPYC active site [11]. Like all mammalian TrxR isoforms, S. mansoni TGR is a selenoprotein with a carboxyl-terminal GCUG active site motif, where “U” is selenocysteine (Sec). Sec is a highly reactive amino acid that gives unique properties to selenoproteins [12]. It is encoded by a dedicated UGA codon in the selenoprotein mRNA and is recoded from translational termination to Sec insertion by a translation machinery utilizing a specialized structural element in the 3′-untranslated region, the SECIS element, which is also found in the mRNA of S. mansoni TGR [11].

Given the importance of cellular redox systems and the biochemical differences between the redox metabolism of S. mansoni and its human host, we hypothesized that TGR could be an essential parasite protein and a potentially important drug target. To test this hypothesis, we used RNA interference (RNAi), characterized the recombinant selenoprotein, and screened inhibitory compounds, including two established antischistosomal drugs that are no longer commonly used, potassium antimonyl tartrate (PAT) and oltipraz (OPZ).

## Methods

### Parasite Preparation

Percutaneous infection of outbred mice (NIH Swiss or Swiss-Webster) with S. mansoni cercariae (NMRI strain) obtained from infected Biomphalaria glabrata snails, perfusion of adult worms (6–7 wk) and juvenile worms (23 d) and preparation of schistosomula from cercariae were as described [13]. This study was approved by the Institutional Animal Care and Use Committee of Illinois State University (08–2002; Department of Health and Human Services animal welfare assurance number A3762–01).

### Recombinant Sec TGR Expression and Purification

A bacterial-type SECIS element was fused to the TGR open reading frame [14] using PCR with the following oligonucleotides primers: forward 5′-catATGCCTCCAGCTGATGGAAC-3′ and reverse 5′-TCGCCAACGACTCCAATTATTAGCCAACGTCCAGACGTGGTTAGCAATTGGATACGCGGGcagctg-3′. The entire TGR ORF plus the SECIS element was subcloned into pET-24a using NdeI/SalI to release the insert. Recombinant TGR was subsequently expressed in the Escherichia coli strain BL21(DE3) in the presence of pSUABC [14] in LB medium supplemented with 20 μM flavin adenine dinucleotide, and otherwise conditions for optimal selenoprotein expression were followed as described [15]. Cultures were centrifuged, lysed by alternative freeze-thaw, resuspended in TE buffer and supplemented with 20 μM flavin adenine dinucleotide. The sample was sonicated and cellular debris pelleted at 25,000*g* at 4 °C for 25 min. The supernatant was collected and filtered through a 0.45 μm filter and brought to a final concentration of 200 mM NaCl. TGR was then purified on an adenosine 2′,5′-diphosphate agarose (Sigma, http://www.sigma-aldrich.com) column equilibrated with TE buffer. The column was washed with 50 ml of TE. TGR was eluted with 1 mM NADPH in TE essentially as described [14,15]. The TGR sample (1/50 volume cell culture) was applied to a 2.5 ml column, which was then washed with 50 ml of TE after which TGR was eluted in ten 1 ml fractions of 1 mM NADPH in TE. Protein purity was >95% as determined by SDS-PAGE, and TGR concentration was determined from the flavin adenine dinucleotide absorption (ɛ_463_ = 11.3 mM^−1^cm^−1^). The pure protein was dialyzed against PBS and stored at −80 °C. Overall yield was ~10 mg of purified protein per liter of culture.

### TGR Enzymatic Assays

Assays were performed at 25 °C in 0.1 M potassium phosphate (pH 7.4), 10 mM EDTA using 100 μM NADPH unless otherwise stated. The insulin assay and the 5,5′-dithiobis(2-nitrobenzoic acid) (DTNB) assays were used to determine the TrxR activity of TGR. The DTNB assay [16] contained 3 mM DTNB, and the initial increase in A_412_ during the first 3 min was recorded upon enzyme addition. One enzyme unit in the DTNB reduction assay was defined as the NADPH-dependent production of 2 μmol of 2-nitro-5-thiobenzoic acid per minute using ɛ_412 nm_ = 13.6 mM^−1^ cm^−1^. The insulin assay [17] mixture contained 1 mg/ml insulin and 10 μM schistosome Trx1 [18], and enzyme activity was monitored by observing the decrease in A_340_ during the first 3 min due to consumption of NADPH (ɛ_340 nm_ = 6.22 mM^−1^ cm^−1^). GR activity was determined with 100 μM GSSG [19]. Grx activity was determined by monitoring the consumption of NADPH at 340 nm during the GSH-dependent reduction of 8 mM β-hydroxyethyl disulfide (HED) using 1 mM GSH coupled with of 0.6 units of yeast GR (Sigma) [20].

The inhibitory action of both PAT and OPZ was addressed by varying substrate and inhibitor concentrations as follows: DTNB 50, 100, 400, 1000 μM; GSSG 10, 25, 50, 100 μM; PAT 10, 30, 75 nM; OPZ 25, 50, 100 μM. The inhibitory constants (*K*_i_) were determined using Equation 1 [21]:

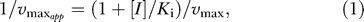
where *v*_max_app__ is the apparent *v*_max_ at different inhibitor concentrations and corresponds to the value of the 1/*v* intercept on the reciprocal plot.

Initial inhibitor screens were conducted in 200 μl volumes in 96 well plates with 50 μM inhibitor, 100 μM NADPH, and 5 nM TGR in 0.1 M potassium phosphate (pH 7.4), 10 mM EDTA with a preincubation step of 15 min. Aurothioglucose was obtained from Research Diagnostics (http://www.researchd.com); aurothiomalate, menadione, methylene blue, naphthazarin, plumbagin, praziquantel, PAT, and safranin were from Sigma; OPZ was a gift from Dr. D. Cioli; auranofin (AF) was a gift from Dr. C.F. Shaw; and all other compounds were prepared as described in the references in the appropriate Tables. Upon addition of another aliquot of NADPH (final NADPH concentration of 100–200 μM) and 3 mM DTNB the activity was measured during the first 3 min by monitoring the A_412_. Residual activity was compared to controls incubated with equal volumes of inhibitor solvent. Compounds exhibiting >85% inhibition were analyzed in detail to determine IC_50_ values using the assays described above and including a 15 min preincubation step of TGR plus inhibitor and 100 μM NADPH. All assays were done in triplicate.

### Kinetic Analysis

TGR activity with the following substrates was determined: GSSG, GSH, HED, S. mansoni Trx1 [18], DTNB, H_2_O_2_, t-butyl hydroperoxide, L-cysteine, sodium selenite, lipoic acid, lipoamide, alloxan, dehydroascorbic acid, and ubiquinone by monitoring the oxidation of NADPH. In all assays the reactions were performed in 1 ml 0.1 M potassium phosphate (pH 7.4), 10 mM EDTA using 100 μM NADPH and 20 nM TGR. Kinetic parameters were determined using Lineweaver–Burk plots using KaleidaGraph 4 (Synergy Software, http://www.synergy.com) least squares best fit of data and varying concentrations of one substrate while maintaining the concentration of other substrates. In the TrxR assays, substrate concentrations ranged from 2.5 to 200 μM NADPH and from 0.25 to 15 μM S. mansoni Trx1 or from 100 to 4,000 μM DTNB. To determine kinetic parameters for GR activity of TGR, substrate concentrations varied from 7.5 to 100 μM GSSG and from 10 to 100 μM NADPH. Grx activity was determined by using 25 to 4,000 μM HED, 10 to 100 μM GSH, and 1 to 100 μM NADPH. All assays were done in triplicate.

### Inhibitor Studies on Cultured Worms

AF was dissolved in DMSO and added to freshly perfused worms in DMEM to 10 μM. Control worms were treated with equal amounts of carrier. Worms were subsequently observed for unpairing, motility, and mortality, and were collected at the indicated time intervals for analysis. Worms were homogenized by sonication in PBS and homogenates were assayed for TrxR and GR activities as described above. Activities of the control enzymes GSH peroxidase (GPx) and lactate dehydrogenase (LDH) were determined using published methods [22,23] with 1 mM sodium azide, 3.0 units of yeast GR (Sigma), 1 mM GSH, 0.02 mM DTT, 0.0007% (w/v) H_2_O_2_, and 100 μM NADPH in the GPx assay and 10 μM sodium pyruvate and 100 μM NADPH in the LDH assay. A replicate experiment was performed in which the worms were homogenized in 1% picric acid and the ratio of GSH:GSSG was determined as described [24]. Each assay was done in triplicate and each experiment was done three times.

### Inhibitor Studies on Infected Mice

Mice (14-wk-old females; C57BL/6 in experiment 1, NIH-Swiss in experiment 2) infected with 60 S. mansoni cercariae were injected intraperitoneally with 6 mg/kg of AF twice daily for 9 d, beginning 7 wk post infection. Infected control mice were injected with equal amounts of carrier. Mice were perfused 1 wk after the final dose of AF and worm burdens were determined.

### RNA Silencing

A 500-nucleotide fragment (bp 1364–1866) of S. mansoni TGR was amplified by PCR (forward primer 5′-CTATTTGGCTAGACGTCTGT-3′ and reverse primer 5′-AATACAGTTTCCTTCCCGTT-3′) and cloned into PCRII-TOPO vector (Invitrogen, http://www.invitrogen.com). The resulting construct was linearized using XhoI (for SP6 RNA polymerase transcription) and SacI (for T7 RNA polymerase transcription). RNA transcription was conducted according to manufacturer's instructions (Ambion, http://www.ambion.com). A 280-bp, nonschistosome dsRNA used for irrelevant dsRNA negative controls was synthesized from the PCRII-TOPO vector using T7 or SP6 RNA polymerases as described above.

Cercariae were mechanically transformed to schistosomula by vortexing, and bodies were separated from tails by Percoll gradient centrifugation as described [13]. Immediately after transformation, TGR dsRNA or irrelevant dsRNA (each at 54 μg/ml) was added to ~500 schistosomula in 50 μl water followed by incubation at 37 °C for 30 min. After addition of 250 μl of RPMI-1640 medium, 300 U/ml penicillin, and 175 μg/ml streptomycin, parasites were incubated at 37 °C in 5% CO_2_ atmosphere for 4 d. Reduced O_2_ tension was obtained by filtering media and purging with 95% N_2_, 5% CO_2_ for 20 min before use. Parasites were cultured during the course of the experiment in the appropriate gaseous mix at 37 °C in a sealed modular incubator chamber (Billups-Rothenberg, http://www.brincubator.com) flushed with the same gaseous mix. On day 2 of the experiment, 50 μl of fresh media was added. Schistosomula were assessed daily for viability by microscopy. For each time point at least 1,500 parasites were scored as alive or dead in each experiment (three replicates of 500 each), and each experiment was repeated three times.

Total RNA was harvested from parasites after the course of an experiment by TRI reagent, following the manufacturer's instructions (Sigma). Complementary DNA was synthesized using 1 μg of RNA, 1 μl of oligo dT (500 μg/ml), and 1 μl of 10 mM dNTP mix in a 10 μl reaction. The mixture was heated to 65 °C for 5 min and quickly chilled on ice. Then 4 μl of first-strand buffer, 2 μl of 0.1 M dithiothreitol, and 1 μl of RNaseOut (Promega, http://www.promega.com) were added to the mixture. The mix was incubated for 2 min and then 1 μl of Thermoscript reverse transcriptase (200 U) (Invitrogen) was added and incubated for 50 min at 42 °C. The reaction was heated at 70 °C for 15 min to inactivate the enzyme. The resulting single-stranded cDNAs were used as templates in PCR reactions using gene-specific primers to amplify TGR (forward 5′-CTATTTCCGTAGACGTCTGT-3′ and reverse 5′-AATACAGTTTCCTTCCCGTT-3′) or GAPDH (forward 5′-GTTTTGGTCGTATCGGGAGA-3′ and reverse 5′-ATGCGTTAGAAACCACGGAC-3′).

### Cytotoxicity Assay

Cytotoxicity assays were performed using sulforhodamine B to determine cellular protein content as described [25]. Briefly, myeloma cell line SP2/0 was cultured in 96-well microtiter plates containing 0.2 ml of RPMI-1640 per well at a cell density of 1,000 per well at 37 °C in 5% CO_2_. Cells were treated with drug concentrations (or drug carrier alone) and exposure times as indicated. After treatment, cells were fixed with 10% TCA at 4 °C for 1 h. Fixed cells were rinsed to remove fixative and then stained in 0.4% (w/v) sulforhodamine B (Sigma) in 1% acetic acid for 30 min. After washing with 1% acetic acid and dye extraction in 10 mM Tris (pH 10.5), plates were read at A_564nm_. The A_564nm_ of drug-treated cells was compared to carrier-only-treated cells. The treatments were done in triplicate and the experiments repeated three times.

### Statistical Analysis

The significance of the reduction in worm burdens after AF treatment was determined by two-tailed Student *t*-test.

## Results

### Biochemical and Kinetic Analysis of Recombinant S. mansoni TGR

Because it is a selenoprotein, it is not possible to directly express recombinant schistosome TGR in Escherichia coli. However, after we added a bacterial-type selenocysteine insertion sequence element to the open reading frame we successfully expressed TGR, using the strategy previously employed for production of mammalian TrxR [14,15]. The purified recombinant TGR demonstrated substantial activity with a broad range of substrates, combining the characteristic activities of mammalian TrxR, GR, and Grx. The specific activities were 10.2 U/mg with DTNB, 2.2 U/mg with Trx (coupled to insulin reduction), 7.2 U/mg with GSSG, and 9.9 U/mg in a Grx assay coupled to HED. In addition, TGR could also reduce sodium selenite, H_2_O_2_, tert-butyl hydroperoxide, alloxan, lipoic acid, and lipoamide. It could not, however, reduce dehydroascorbic acid or ubiquinone. Table 1 summarizes the kinetic parameters of the enzyme with these different substrates.

**Table 1:**
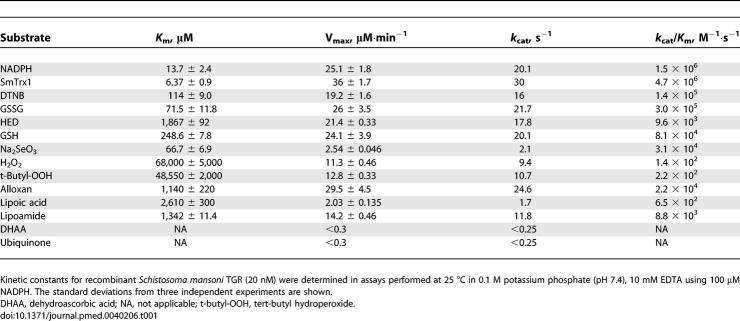
Kinetics of Recombinant S. mansoni TGR with Different Substrates

The enzymatic properties of recombinant parasite TGR differed from those of mammalian TGR [26]; schistosome TGR had greater GSSG reduction activity relative to Trx reduction (GSSG:Trx ratio of 4:1), whereas native mouse TGR had the reverse proportions (1:2), and S. mansoni TGR had 6-fold higher Grx activity than mouse TGR (Table 2). The activity and kinetic constants of recombinant S. mansoni TGR were similar to those reported for mammalian TrxR [26] and for human GR (Table 2) [27,28], although its activity was lower than that of human GR.

**Table 2:**
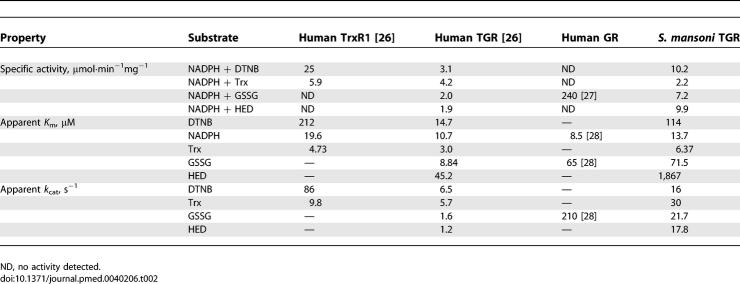
Comparison of the Kinetic Properties of Human TrxR1, TGR, and GR to Recombinant S. mansoni TGR

### Inhibition Studies on Recombinant S. mansoni TGR

In order to assess the potential for inhibition of TGR we screened several structurally diverse series of compounds (Figure S1) known to act as inhibitors of disulfide reductases from various organisms. Screening was performed with a TGR assay using DTNB, including a 15 min preincubation of recombinant enzyme, inhibitor, and NADPH before addition of substrate (Table 3). Compounds exhibiting >85% inhibition in the initial screen were analyzed further to determine IC_50_ values for GR, TrxR, and Grx activities of TGR using the appropriate assays with a 15 min preincubation (Table 4). In order to determine if compounds showed selective inhibition of TGR compared to human TrxR and GR, the IC_50_ values for TGR were compared to those of human TrxR and GR either reported in literature or determined here (Table 4).

**Table 3:**
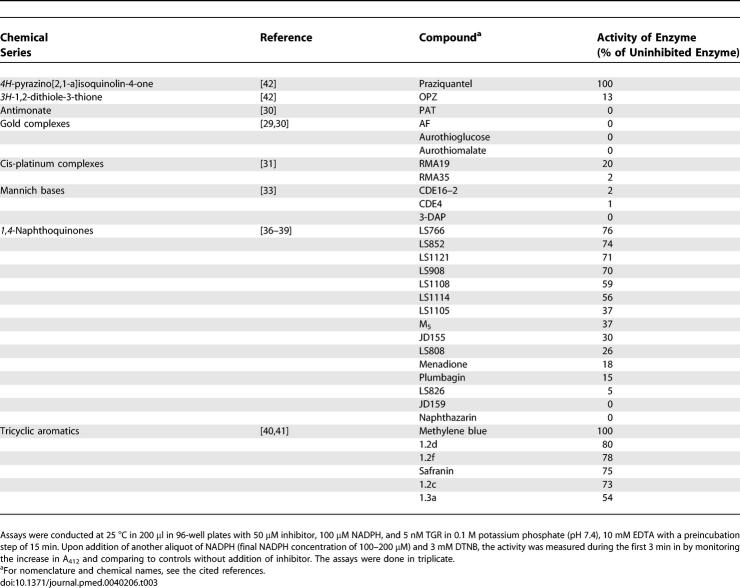
Primary Screen of NADPH-Dependent Oxidoreductase Flavoprotein Inhibitors and Antischistosome Drugs as Inhibitors of S. mansoni TGR

**Table 4:**
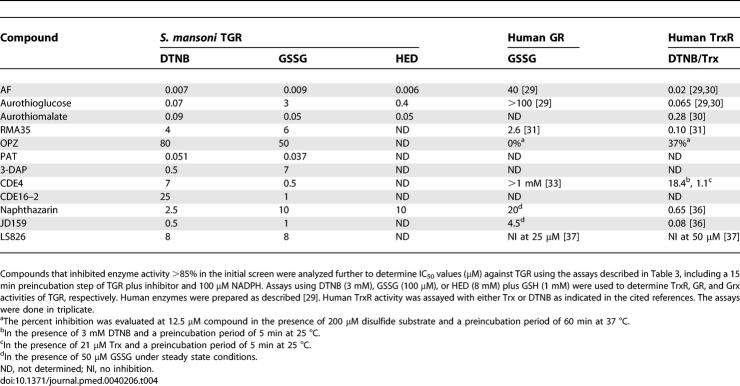
IC_50_ Values of TGR Inhibitors with 15-Minute Preincubation Assays

The gold complexes AF, aurothioglucose, and aurothiomalate, which are efficient inhibitors (i.e., with low *K*_i_/IC_50_ values) of mammalian TrxR [29,30], were found also to be efficient TGR inhibitors. AF was the most potent inhibitor of TGR, with IC_50_ values in the low nanomolar range. Aurothioglucose had a less inhibitory effect on the reduction of GSSG and Grx activity (HED-coupled assay) than on the reduction of DTNB, whereas the other two gold compounds inhibited to a similar extent all three principal activities of TGR (Table 4). This indicates that the Grx activity, presumably catalyzed by the N-terminal Grx domain of the enzyme [11], could be dissociated from the other activities of the enzyme, which should be catalyzed by the C-terminal GCUG motif. This interpretation is corroborated by the fact that a C-terminally His-tagged form of the enzyme lacking the Sec residue was previously found to lack TrxR or GR activity, while still supporting Grx activity [8].

Platinum drugs (e.g., RMA19 and RMA35) are also irreversible inhibitors of mammalian TrxR [31,32]. RMA35, but not RMA19, was here a potent inhibitor of TGR; it had the opposite effect with human TrxR [31], indicating that specific structural features can be exploited for the design of selective TGR inhibitors.

The most active Mannich base, inhibitors of plasmodial and kinetoplastid flavoenzymes [33,34], was 3-DAP, with low micromolar IC_50_ values against TGR. The naphthoquinones juglone [35] and naphthazarin [36] and several of their derivatives are subversive substrates of human TrxR. Among the naphthoquinones, LS826 [37] behaved as a selective TGR inhibitor, with an IC_50_ of 8 μM, but as a poor inhibitor of the human enzymes. While the GR inhibitor M_5_ [38,39] had no effect on TGR, naphthazarin and its derivatives JD155 and JD159 [36] inhibited TGR, but not selectively. Tricyclic aromatics, which are inhibitors of Plasmodium falciparum growth and human GR [40,41], were inactive as inhibitors of TGR.

**Figure 2:**
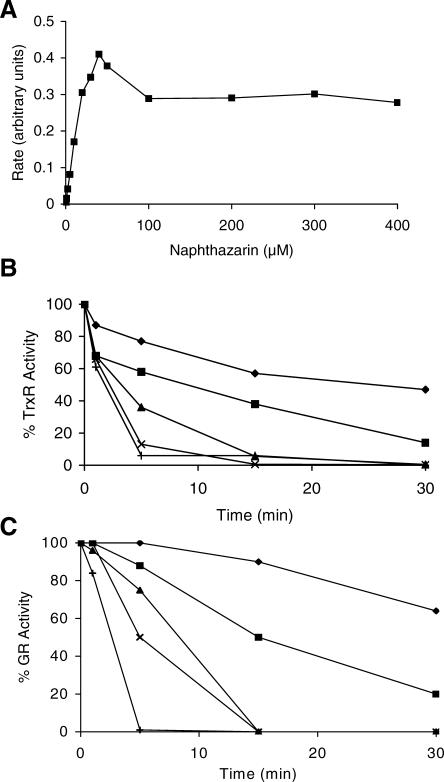
Naphthazarin Is a Substrate and Inhibitor of TGR (A) Activity of recombinant S. mansoni TGR with naphthazarin as a substrate was determined by NADPH consumption under steady-state conditions. (B) Time-dependent inhibition of TrxR activity of TGR by naphthazarin with DTNB as a substrate. Concentrations of naphthazarin are 0.5 μM (♦), 1 μM (█), 2.5 μM (▴), 5 μM (×), and 10 μM (+). (C) Time-dependent inhibition of TGR activity with GSSG. Concentrations of naphthazarin are 0.5 μM (♦), 1 μM (█), 2.5 μM (▴), 5 μM (×), and 10 μM (+).

Similar to the interactions of mammalian TrxR with several quinones [35], inhibitory compounds may function as both substrates and inhibitors (i.e., subversive substrates) of TGR. The interaction of schistosome TGR with naphthazarin was found to exhibit this type of effect, with naphthazarin both acting as substrate (Figure 2A) and leading to time-dependent inhibition (Figure 2B and 2C).

**Figure 3:**
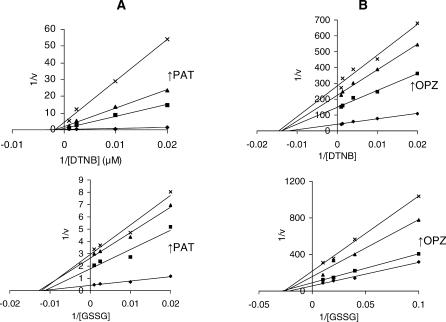
Inhibition of S. mansoni TGR by PAT and OPZ Reciprocal plots showing inhibition of the TrxR and GR activities of TGR by PAT (A) and OPZ (B). Both are noncompetitive inhibitors, altering the V_max_ while the *K*_m_ for substrates remains constant. The apparent V_max_ for various concentrations of inhibitors were used to calculate *K*_i_ values. Concentrations of PAT are no drug (♦), 10 nM (), 30 nM (▴), and 75 nM (×); and of OPZ are no drug (♦), 50 μM (), 75 μM (▴), and 100 μM (×). Concentrations of DTNB ranged from 50 to 1,000 μM (TrxR activity) and of GSSG from 10 to 100 μM (GR activity).

We examined three antischistosomal drugs [42,43], praziquantel, OPZ, and PAT. Both PAT and OPZ were here efficient, noncompetitive inhibitors of TGR (Figure 3); the *K*_i_ values (± standard deviation) for PAT were 4.86 ± 0.46 nM (TrxR activity) or 7.22 ± 4.96 nM (GR activity), and for OPZ were 18.1 ± 1.1 μM (TrxR activity) or 33.8 ± 6.0 μM (GR activity). The widely used antischistosomal drug praziquantel displayed no inhibitory activity against TGR.

**Figure 4:**
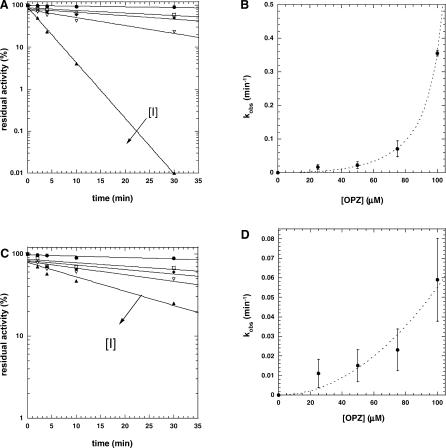
Time-Dependent Inactivation of S. mansoni TGR by OPZ The time dependency of inactivation of *S*. *mansoni* TGR (15 pmol/μl) was revealed by determining the residual GR (A) and TrxR (C) activities of enzyme in the presence of 100 μM NADPH and inhibitor at 0, 2, 4, 10, and 30 min incubation periods. OPZ concentrations were 0 (•), 25 (□), 50 (♦), 75 (Δ), and 100 (▴) μM. All incubation mixtures included 2% final DMSO. The *k*_obs_ data as a function of inhibitor concentration from the GR (B) or TrxR assay (D) versus OPZ concentrations followed a polynomial equation (not shown), suggesting that more than one binding site of OPZ is involved in the presence of different enzyme species of reacted and unreacted enzymes in mixture during the course of the inactivation process. The error bars in (B) and (D) represent ± the standard errors of the *k*_obs_ values estimated separately at each inhibitor concentration, as obtained from nonlinear regression analysis.

Inactivation of S. mansoni TGR by OPZ followed pseudo-first order reaction kinetics. A semi-logarithmic plot of the fraction of noninhibited enzyme activity ln(*v*_i_/*v*_0_) versus incubation time yielded linear curves with increasing slopes, equivalent to the apparent rate constant of irreversible inhibition (*k*_obs_) in GR (Figure 4A) and in TrxR (Figure 4C) assays. The *k*_obs_ values determined for S. mansoni TGR inactivation by OPZ ranged from 16.3 × 10^−3^ min^−1^ to 355.5 × 10^−3^ min^−1^ for GR activity and from 11.0 × 10^−3^ min^−1^ to 58.9 × 10^−3^ min^−1^ for TrxR activity, within the log-linear range of the inhibition curve. When the derivation described by Kitz and Wilson [44] for irreversible inactivation was applied to the experimental data, only at low inhibitor concentration, the secondary plot expressing *k*_obs_ as a function of inhibitor concentration followed Equation 2:

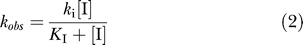
where *K*_I_ represents the dissociation constant of the inhibitor, and *k*_i_ is the first-order rate constant for irreversible inactivation. In both secondary plots (Figure 4B and 4D) showing inactivation of *S*. *mansoni* TGR at low OPZ concentrations, a rough estimation of *k*_i_, the resulting half-time value (*t*_1/2_), and *K*_I_, were 0.04 min^−1^, 17.3 min, and 32.8 μM in the GR assay, and 0.06 min^−1^, 11.5 min, 140 μM in the TrxR assay, respectively. The resulting second-order rate constant values of *k_i_*/*K*_I_ were 7.1 M^−1^ s^−1^ and 20.3 M^−1^ s^−1^ for inactivation of, respectively, GR and TrxR activities by OPZ. At higher inhibitor concentrations, the curves were not hyperbolic, but became polynomial, suggesting that more than one binding site of OPZ is involved in the presence of reacted and unreacted enzymes species in mixture. A double reciprocal replot of *k*_obs_ *versus* [I] did not fit to a linear relationship (unpublished data). In close accord with observations made in recent studies on the inactivation of human glutathione reductase [45] or of human thioredoxin reductase [31], the inactivation of S. mansoni TGR by OPZ likely involves distinct enzyme populations, i.e., the enzyme species reduced at the flavin for inhibitor reduction, or the major two-electron reduced form with a dithiol in the active site(s) for alkylation. Although covalent inactivation of the enzyme was observed in both GR and TrxR assays, the kinetics of inactivation indicated that OPZ interacts in a complex way with the multifunctional enzyme *S*. *mansoni* TGR.

### Inhibitor Studies on Cultured Worms

**Figure 5:**
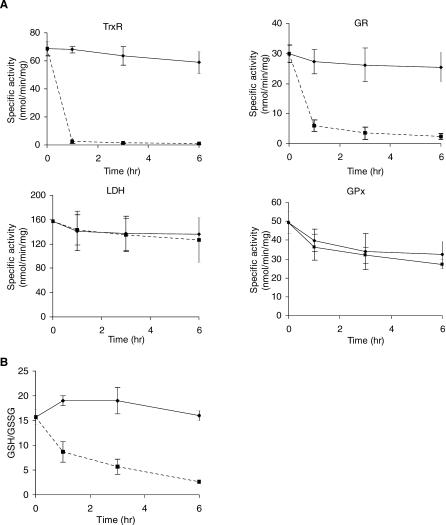
The Activity of Auranofin against Cultured Schistosoma mansoni Each point is the average of three independent experiments ± the standard deviation. (A) Specific enzyme activities of TrxR, GR, LDH, and GPx in worm homogenates from control worms (solid lines) and worms treated with 10 μM AF (dashed lines). (B) Ratio of GSH:GSSG in worm homogenate from control worms (solid line) and worms treated with 10 μM AF (dashed line). Each assay was done in triplicate and each experiment was done three times. The error bars show the standard deviation of the three replicate experiments.

Because of the potent effect of AF on recombinant S. mansoni TGR activity, we also analyzed its effect on worm pairing (female worms are present in copula in the male gynocophoral canal, and worms will remain paired for several days in optimal axenic culture) and worm viability (as evidenced by muscle contraction and parasite movement). Incubating worms with 10 μM AF resulted in unpairing of male and female worms after 1 h, 83% mortality after 6 h, and 100% mortality after 9 h of treatment; control worms remained paired and active throughout the treatment. When compared to control worms, TrxR and GR activities of TGR in worm homogenates were nearly 100% inhibited after 1 h of treatment with 10 μM AF (Figure 5A). In contrast, the activities of control enzymes, LDH and GPx, showed no significant deviation from controls (Figure 5A). The ratio of GSH:GSSG was also determined at each time point. The control worms displayed a relatively constant ratio of ~18:1, while in AF-treated worms a gradual oxidation of glutathione was detected as early as 1 h of AF treatment. After 6 h of treatment the GSH:GSSG ratio had decreased by 85% to 2.6:1 (Figure 5B).

**Figure 6:**
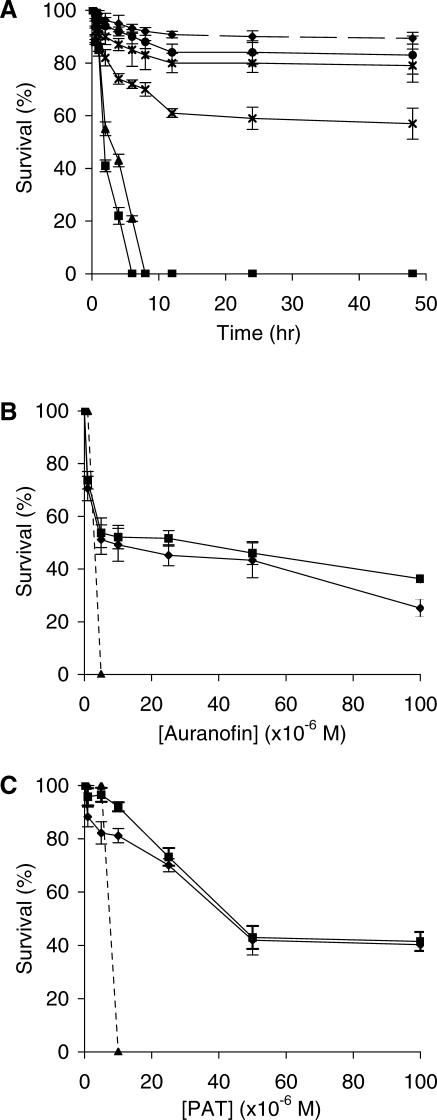
Survival of Schistosomula, Adult Worms, and Myeloma Cells in the Presence of Auranofin or Potassium Antimonyl Tartrate (A) Cultured schistosomula treated with 0 μM AF (dashed line); AF 0.5 μM (♦); 1 μM (*); 2 μM (x); 5 μM (▴); 10 μM (). (B) Survival of adult Schistosoma mansoni worms at 24 h (dashed line), myeloma cells at 24 h (); and myeloma cells at 5 d (♦) at given concentrations of AF. (C) As in (B) in the presence of given concentrations of PAT. Each treatment was done in triplicate and each experiment was done three times. Error bars show the standard deviation of the three replicate experiments.

Likewise, larval, skin stage parasites, and juvenile liver stage parasites (unpublished data) are killed in less than 10 h by AF at concentrations as low as 5 μM (Figure 6A). For comparison, a mammalian cell line tolerated AF at concentration as high as 100 μM for 5 d, while 5 μM AF led to adult worm death in 24 h (Figure 6B). A similarly large differential in toxicity between adult worms and mammalian cells for PAT was also found (Figure 6C).

### In Vivo Inhibition Studies

To further investigate the potential of TGR as a drug target, we analyzed the possible effects of AF treatment on the survival of worms in mammalian hosts. In preliminary studies, mice infected with S. mansoni were administered 6 mg/kg AF twice daily for 9 d, which is a safe dose for healthy mice [46], beginning 7 wk after infection (when adult worms are present in the mesenteric venules) followed by a 1 wk rest. Mice were then perfused and worms were collected and counted. In two independent experiments utilizing different mouse strains, the AF-treated mice had a 59% and 63% decreases in worm burden compared to control mice (Table 5).

**Table 5:**
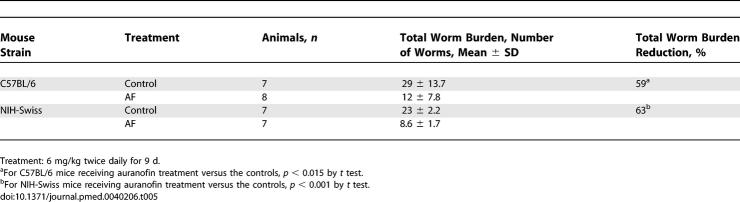
Effect of AF Treatment in Mice Experimentally Infected with S. mansoni

### RNAi Silencing of TGR

**Figure 7:**
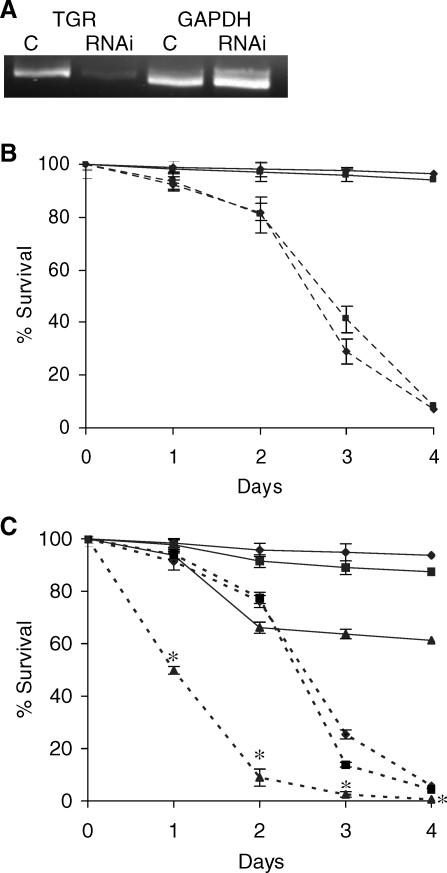
Survival of Schistosomula after RNAi Silencing of TGR Expression (A) Qualitative measure of TGR transcripts by reverse transcription PCR. The abundance of TGR mRNA was greatly reduced by dsRNA treatment while the control gene *GAPDH* was unaffected. (B) Parasites were cultured in the presence of double-stranded TGR RNA (dashed lines) or of double-stranded irrelevant RNA (solid lines) in 20% O_2_ (♦) or 0% O_2_ (). A 500-nucleotide fragment (bp 1364–1866) of S. mansoni TGR cloned into PCRII-TOPO vector was used to transcribe TGR dsRNA. Irrelevant, nonschistosome dsRNA used for negative controls was synthesized from the PCRII-TOPO vector using T7 and SP6 RNA polymerases. (C) Combination of RNAi and drug treatments. Solid lines are irrelevant dsRNA treatments and dashed lines are dsTGR treatments; no additions (♦), 2 μM praziquantel () and 2 μM AF (▴). At each time point, data comparing the combination treatment (2 μM AF + TGR dsRNA) to either 2 μM AF or TGR dsRNA alone were statistically significant (*p < 0.004). In each experiment for each time point at least 1,500 parasites were scored as alive or dead (three replicates of 500 parasites). The error bars represent the standard deviations of three independent experiments.

**Figure 8:**
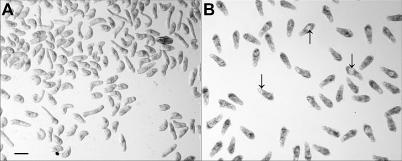
Photomicrographs (100×) of Irrelevant dsRNA-Treated Schistosomula (left image) and TGR dsRNA-Treated Schistosomula (right image) after Three Days of Treatment All organisms in the left image are alive; parasites have different shapes, elongated, contracted, and curved during movement. In the right image, all of the parasites are dead and have roughly the same shape (no movement) and internal vacuoles (arrows). The bar represents 250 μm.

In order to further analyze the importance of TGR for worm survival, we incubated schistosomula (larval parasites) with double-stranded TGR RNA to silence TGR expression. After 2 d of TGR dsRNA treatment, TGR activity (using DTNB as substrate) was reduced by 35% in parasites in aerobic culture (20% O_2_) and after 3 d of TGR dsRNA treatment, TGR activity was reduced by 61% or 63.5% in parasites cultured anaerobically or aerobically, respectively. A marked decrease in TGR mRNA after 3 d of treatment was also seen (Figure 7A). The RNAi silencing of TGR led to substantial decreases in parasite survival; approximately 92% of dsRNA-treated parasites were dead after 4 d of treatment both in aerobic and anaerobic conditions (Figure 7B). Control parasite survival was over 95% in both aerobic and anaerobic growth conditions. After 3 d, TGR dsRNA-treated parasites had darker bodies with internal vacuoles and did not move, while all irrelevant dsRNA-treated parasites showed contractile movement and had clear bodies (Figures 8 and Figure S2). Treatment of cultured parasites with sublethal levels of AF and TGR dsRNA showed an additive effect significantly accelerating the killing of schistosomula compared to RNAi or AF treatments alone (Figure 7C). Schistosomula treated with both TGR dsRNA and 2 μM AF had 49.8% ± 2.8 % survival after 1 d compared to 91.3% ± 1.1% for RNAi alone and 93.7% ± 1.1% for 2 μM AF alone (*p* < 0.001), and after 2 d survival in the combination treatment was 8.9% ± 1.5% compared to 66.1% ± 1.4% for 2 μM AF alone and 76.1% ± 3.2% for RNAi alone (*p* < 0.001), with similarly significant results over the remainder of the 4 d time course. Praziquantel had no effect alone or in combination with RNAi.

## Discussion

In this study we have demonstrated that TGR is an essential protein for the survival of S. mansoni and that it meets all the major criteria of an important target for antischistosomal chemotherapy development. Silencing of TGR expression by RNAi lead to rapid parasite death, and auranofin, a specific chemical inhibitor of TGR, provides partial parasitological cures of infected mice. We have screened a number of TGR inhibitors and identified potential lead compounds for novel drug development. Furthermore, we demonstrated that TGR was likely a key target of some earlier therapies.

It has been suggested that antioxidants play an important role in protecting adult S. mansoni worms from immune attack by the host [47]. Recent studies indicate that the enzymatic antioxidant pathway in S. mansoni is uniquely dependent on TGR [11]. In the parasite, TGR completely replaces more specialized TrxR and GR orthologs and, therefore, TGR functions in reducing both Trx and GSSG. This may lead to a bottleneck effect that should make TGR an attractive drug target. The results presented here strengthen this hypothesis. Recombinant S. mansoni TGR was here shown to possess substantial TrxR, GR, and Grx activity, with rates comparable to those reported for native TGR purified from mouse testis [26] and more than 40-fold more active than recombinant mouse TGR [48]. When S. mansoni TGR was first cloned, we proposed that the enzyme was a selenoprotein with a penultimate Sec residue, and that TGR should have enzymatic activity dependent on the C-terminal Sec-containing motif in analogy to mammalian TrxR and TGR isoforms [11]. This study, in which the enzyme was expressed as a selenoprotein, showed that hypothesis to be correct. It should be noted, however, that the Grx activity of the enzyme was sustained without the C-terminal motif, as shown here and previously [11].

Characterization of the parasite TGR revealed enzymatic properties that differed from those of mammalian TGR, TrxR, and GR. TGR was also identified as a multifunctional oxidoreductase with a remarkably wide substrate specificity, capable of directly reducing peroxides, selenium-containing compounds, and several important low-molecular-weight antioxidants, as well as Trx, DTNB, GSSG, and GSH-HED. However, TGR was unable to reduce dehydroascorbic acid or ubiquinone, compounds that are reduced by mammalian TrxR1 [49,50]. These findings show that TGR is likely to serve multiple functions in the parasite and is functionally distinct from the TrxR and GR orthologs of the human host. These substrate preferences might possibly be exploited for future TGR-directed anti-schistosome drug design.

Several additional differences exist between schistosome TGR and mammalian TGR, TrxR1, TrxR2, or GR. In mammals, TGR is thought to serve a highly specialized function with expression restricted primarily to the testis [26,51]. Both TrxR1 and TrxR2 isoenzymes have a nearly ubiquitous distribution in mammalian tissues; TrxR1 is predominantly a cytoplasmic enzyme [52], while TrxR2 is mitochondrial [53]. GR is also widely distributed and is believed to be primarily cytoplasmic, although at least in yeast an alternatively translated isoform is targeted to the mitochondria [54]. For Schistosoma spp. it seems clear that TGR accounts for the complete combined Trx and GSSG reduction in the parasite. Moreover, schistosome TGR mRNA is alternatively spliced, producing both cytoplasmic and mitochondrial forms of the enzyme (LLC and DLW, unpublished data). Therefore, it appears that schistosome TGR fulfills all of the major functions of the mammalian TGR, TrxR1, TrxR2, and GR orthologs, emphasizing the importance of the parasite protein, and this alone should make it an attractive target for treatment of schistosomiasis. Because redox metabolism in other medically important parasitic platyhelminths is thought to be similar to S. mansoni [55], targeted inhibition of parasite TGR orthologs may also be a useful strategy to develop drugs against other parasitic infections.

The previously used antischistosome drugs PAT and OPZ inhibited recombinant schistosome TGR, suggesting that the enzyme may already have served as a target protein for antischistosomal therapy. The dithiolethione compound OPZ reached Phase III clinical trials for schistosomiasis treatment [42] before it was withdrawn because of adverse side affects [56].

**Figure 9:**
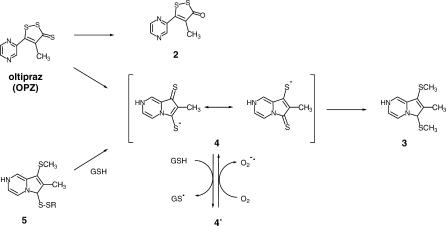
Metabolism of Oltipraz Metabolism of OPZ results in the production of a minor oxo analog (2), and the major dimethylated pyrrolopyrazine (3) through the biological methylation of the intermediate pyrrolopyrazine thione (4).

Although the mechanism of action of OPZ is not well defined, it has been reported to decrease GSH levels in adult S. mansoni worms [57]. Previous studies have shown that OPZ requires metabolite bioactivation both to display potent schistosomicidal activity and inhibition of the GSSG reduction activity [58]. The metabolism of OPZ in humans was investigated and the structures of active metabolites were identified (Figure 9). Metabolism of OPZ results in the production of a minor oxo analog, and the major dimethylated pyrrolopyrazine through the biological methylation of the intermediate pyrrolopyrazine thione. Two general hypotheses have been proposed to explain the mechanisms of OPZ activation [59]. The first suggests that OPZ acts through a bioactive metabolite following reaction with cellular thiols. The generation of the major methylated pyrrolopyrazine thione, an electrophilic intermediate species (structure 4, Figure 9), would lead to alkylation of protein thiols. While the disulfide bond of dithiolethiones is more difficult to reduce than a linear disulfide bond because of its aromatic character, the same pyrrolopyrazines can be obtained both by chemical (use of thiols like GSH) and electrochemical methods from OPZ. The greater reactivity of dithiols compared to monothiols with respect to reactions with dithiolethiones was determined to be due to an intrinsic enhanced reactivity of specific biological dithiol targets [60]. The second hypothesis also involves redox reactions from the major methylated pyrrolopyrazine thione (structure 4, Figure 9) in the presence of oxygen, contributing to a flux of reactive oxygen species [61,62]. Some disulfide intermediates (structure 5, Figure 9) displaying high schistosomicidal activity were shown to act as prodrugs of the pyrrolopyrazine thione (structure 4, Figure 9) [58].

The action mechanism of OPZ against S. mansoni was also extensively investigated. The schistosomicidal activity of OPZ was correlated with glutathione depletion and a 2-fold lower GSSG reduction activity in *S. mansoni,* which was assigned to the action of a metabolite (having the general structure 5, Figure 9) rather than to OPZ itself [58]. Initially, GR was proposed to be responsible for GSSG reduction activity in *S*. *mansoni* and thus to be the target of OPZ. However, because there is no classical GR in this parasite, we checked the activity of OPZ as inhibitor of S. mansoni TGR and/or the prodrug of the species involved in decreased S. mansoni TGR activity in worms. Our results suggest that the antischistosomal effects of OPZ may have at least in part been through inhibition of schistosome TGR and that OPZ should be revisited to improve its toxicological profile.

The first chemotherapy used for schistosomiasis was PAT, a trivalent antimonial that was administered to patients infected with S. haematobium beginning in 1917 [63]. PAT and other trivalent antimonials dominated chemotherapy for schistosomiasis until the introduction of praziquantel in the 1980s. Earlier studies suggested that the antischistosome action of trivalent antimonials is due to the selective inhibition of the parasite glycolytic enzyme phosphofructokinase (PFK) [64,65]. As found here, PAT is a noncompetitive TGR inhibitor with a *K*_i_ value in the low nanomolar range, showing that PAT is by three orders of magnitude a better inhibitor of TGR than of PFK, which has an IC_50_ of 16 μM [65]. Combined with our other results, this suggests that the chemotherapeutic action of PAT is more likely to occur through inhibition of parasite TGR and than of PFK, or that inhibition of both enzymes provides a synergistic antiparasitic affect. We are currently carrying out investigations to test this hypothesis.

Gold compounds may inhibit selenoproteins by targeting the Sec residue, and mammalian TrxR is a selenoprotein that is especially sensitive to such inhibition [29]. Auranofin, aurothiomalate, and aurothioglucose have all been used clinically for the treatment of rheumatoid arthritis [66]. Here we found that gold compounds are also potent inhibitors of schistosome TGR. It should also be noted that, although schistosomes express a GPx with a catalytically active Sec residue [67], GPx activity was not inhibited by AF (Figure 5). Furthermore, the inhibition of TGR by AF in cultured worms greatly reduced the GSH:GSSG ratio and resulted in rapid worm death. These results on cultured worms support the conclusion that TGR should be a prime target for antischistosomal drug therapy. The significant decrease in worm burden in infected mice treated with AF also supports this notion. The concentration of AF used to achieve this affect was well tolerated by the mice in this and previous studies [46], and it was reported previously that mammalian cells have over 95% viability when cultured in 10 μM AF [68]. The reasons for a generally low toxicity toward mammalian cells by AF are not known but could involve low uptake or efficient metabolic defense systems. While mammalian cells and the infected mouse hosts obviously tolerated the dosage of AF used in our studies, the results presented here show that larval, juvenile, and adult parasites are extremely sensitive to killing by AF. It should be noted that the toxic effects of AF occurred in the absence of exogenous oxidative stress (e.g., no added H_2_O_2_ or other oxidant) on the worms.

Partial parasite clearance with AF is not an invalidation of S. mansoni TGR as an essential protein; factors such as the low in vivo bioavailability of AF might be the cause of the incomplete worm clearance. It is also important to emphasize that although the development of a schistosomiasis drug leading to partial cures is not a preferred goal, there are a number of factors associated with schistosome infections to consider. For example, unlike bacterial or protozoal infections, which require 100% cure rates or risk relapse, reduction of worm burdens by 50% would lead to significant decreases in pathology and morbidity associated with schistosomiasis, and no rebound in pathology would occur because adult worms do not multiply in their host. Furthermore, because praziquantel is highly effective and has low toxicity, it is essential to identify new drugs that can be used in combination with it to prevent parasites from developing resistance. Combination therapies may be useful to prevent praziquantel resistance even if only partial worm reductions were affected by the new drug alone. The effectiveness of artemisinin derivatives in combination therapy with praziquantel against schistosomiasis has been demonstrated [3], but concerns remain that the use of artemisinins for multidrug-resistant malaria treatment not be compromised. Our results indicate that praziquantel does not inhibit TGR; therefore, it is reasonable to assume that drugs targeting TGR will have a different mechanism of action than praziquantel. Unlike the more selective action of praziquantel against larval and adult parasites [69,70] and artemisinin against juvenile parasites [71], respectively, inhibition of TGR led to both larval and adult parasite death. Development of drugs targeting parasite TGR may thus provide the opportunity for prophylactic therapies. We propose that the screen for inhibitors of TGR as initiated here be continued and that it may lead to the development of novel drugs with potent antischistosomal effects. In this context it should be noted that the kinetics of inhibition of TGR may be rather complex, as discussed above for OPZ. Further studies are therefore needed with the different inhibitors already identified in the present study for a characterization of their interactions with TGR.

With TGR being a probable important drug target for treatment of schistosomiasis, is it an essential protein for the parasite? The RNAi treatments silencing TGR expression that led to significant decreases in parasite survival indicate that it is. The TGR dsRNA treatment led to a significant decrease in both TGR mRNA and enzymatic activity, while treatment with irrelevant dsRNA had no effect. Genome sequence analysis, although not complete, represents ~95% coverage of the S. mansoni genome. Analysis of the genome sequence indicates that TGR is a single-copy gene (unpublished data) and no other activity is present in worms for Trx and GSH reduction [11]. Parasite death, which occurred in either aerobic or anaerobic conditions, was presumably due to pleiotropic effects of silencing TGR. Furthermore, combined treatment of schistosomula with TGR dsRNA and AF showed significant synergistic effects, suggesting that both act by decreasing TGR activity. Identification of the exact mechanism for the lethal effect of silencing the enzyme requires further experiments, as do the effects of inhibitors, which are not necessarily solely due to loss of enzyme activity, because the inhibited enzyme may also have a gain of function leading to cell death [72].

To conclude, in this report we have provided the first, to our knowledge, comprehensive biochemical analysis of a TGR selenoprotein from any organism*,* showing that it has remarkably wide substrate specificity and may be effectively inhibited by a number of low-molecular-weight compounds. Furthermore, using both inhibitors and RNA silencing we have shown that TGR is an essential schistosome protein, and we believe that our results have validated it as a key drug target for treatment of schistosomiasis.

## Supporting Information

Alternative Language Abstract S1 Translation of the Abstract into Arabic by Ahmed A. Sayed (30 KB DOC)

Alternative Language Abstract S2 Translation of the Abstract into French by Jean Dessolin (29 KB DOC)

Figure S1 Chemical Structures of Compounds Used in This Study as Inhibitors of TGR of S. mansoni
(107 KB PDF)

Figure S2 Photomicrographs (100×) of Irrelevant dsRNA-Treated Schistosomula and TGR dsRNA-Treated Schistosomula after Three Days of Treatment Schistosomula treated with irrelevant dsRNA are shown in the image on the left; those treated with TGR dsRNA are on the right. All organisms in the left image are alive; parasites have different shapes, elongated, contracted, and curved during movement. In the right image, all of the parasites are dead and have roughly the same shape (no movement) and internal vacuoles (arrows). The bar represents 330 μm.
(7.5 MB EPS)

## Abbreviations

AF: auranofin
DTNB: 5,5′-dithiobis(2-nitrobenzoic acid)
GR: glutathione reductase
GPx: glutathione peroxidase
Grx: glutaredoxin
GSH: glutathione
GSSG: glutathione disulfide
HED: β-hydroxyethyl disulfide
LDH: lactate dehydrogenase
OPZ: oltipraz
PAT: potassium antimonyl tartrate
RNAi: RNA interference
Sec: selenocysteine
TGR: thioredoxin-glutathione reductase
Trx: thioredoxin
TrxR: thioredoxin reductase
