# Correction: Thioredoxin Glutathione Reductase from Schistosoma mansoni: An Essential Parasite Enzyme and a Key Drug Target

**DOI:** 10.1371/journal.pmed.0040264

**Published:** 2007-08-28

**Authors:** Angela N Kuntz, Elisabeth Davioud-Charvet, Ahmed A Sayed, Lindsay L Califf, Jean Dessolin, Elias S. J Arnér, David L Williams

Correction for:

Kuntz AN, Davioud-Charvet E, Sayed AA, Califf LL, Dessolin J, et al. (2007) Thioredoxin Glutathione Reductase from Schistosoma mansoni: An Essential Parasite Enzyme and a Key Drug Target. PLoS Med 4(6): e206. doi:10.1371/journal.pmed.0040206

In the supporting information section of this paper, the structures of oltipraz and safranin in Figure S1 were incorrect.

Figure S1 Chemical Structures of Compounds Used in This Study as Inhibitors of TGR of S. mansoni
(407 KB DOC)

