# Supplementary material for: Thioredoxin Glutathione Reductase from Schistosoma mansoni: An Essential Parasite Enzyme and a Key Drug Target
Source: PLoS Med. 2007 Jun 19;4(6):e206. doi: 10.1371/journal.pmed.0040206 (PMC1892040; doi:10.1371/journal.pmed.0040206)

Supplemental Fig. 1. Chemical structures of compounds used in this study as inhibitors of thioredoxin-glutathione reductase (TGR) of *Schistosoma mansoni*.

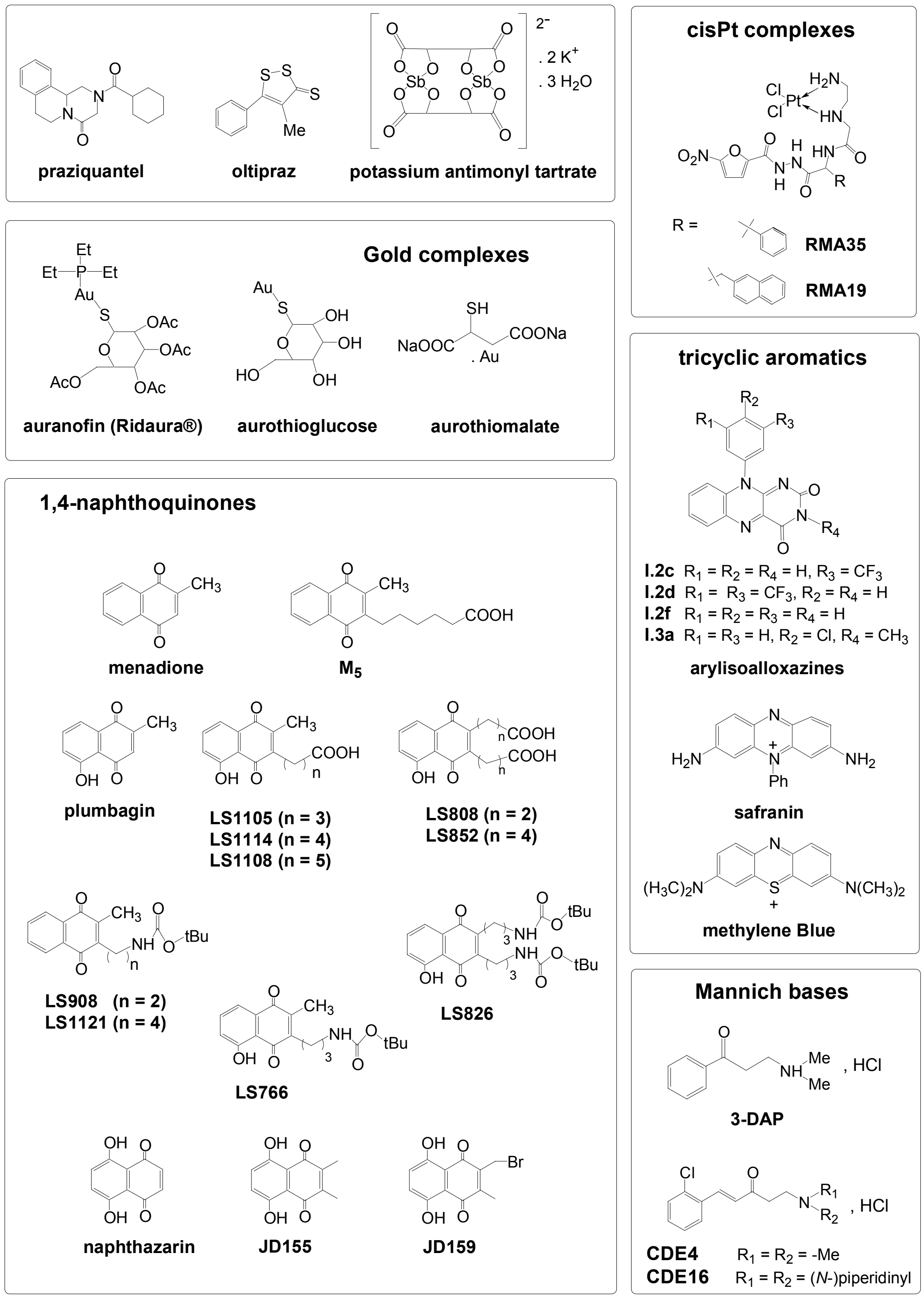

Supplement: Supplementary file 1 [file pmed.0040206.sg001.pdf]
