# Supplementary material for: Thioredoxin Glutathione Reductase from Schistosoma mansoni: An Essential Parasite Enzyme and a Key Drug Target
Source: PLoS Med. 2007 Jun 19;4(6):e206. doi: 10.1371/journal.pmed.0040206 (PMC1892040; doi:10.1371/journal.pmed.0040206)
Supplement: Supplementary file 2 [file pmed.0040206.sg002.pdf]

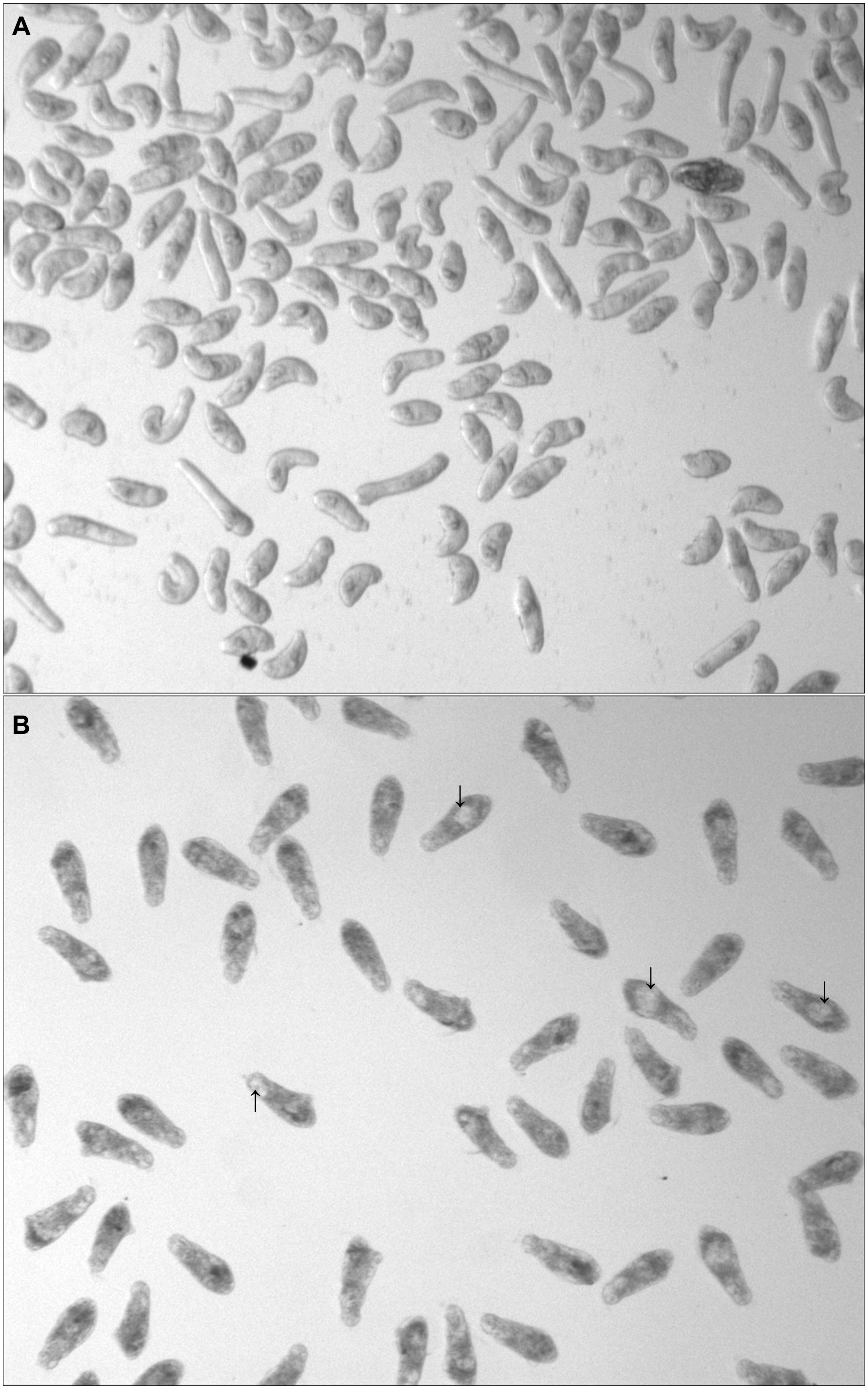
A
B
