# Supplementary material for: Thioredoxin Glutathione Reductase from Schistosoma mansoni: An Essential Parasite Enzyme and a Key Drug Target
Source: PLoS Med. 2007 Jun 19;4(6):e206. doi: 10.1371/journal.pmed.0040206 (PMC1892040; doi:10.1371/journal.pmed.0040206)
Supplement: Supplementary file 3 [file pmed.0040206.sd001.doc]

**الملخص**

**الخلفيه**

يعتبر المرض الناشئ عن الاصابه بطفيل البلهارسيا من امراض المناطق الحاره المدمره والواسعة الانتشار حيث أنه يصيب ما يزيد عن 200 مليون شخص. فعقار (praziquantel)هو العقارالوحيد الواسع الاستخدام في الوقت الراهن ويعالج بهذا العقار قرابة 100 مليون شخص سنويا هذا بإلاضافه الى عدم توافر لقاح لهذا المرض حتى الان. وحيث ان مقاومة الطفيل لهذا العقار امر وارد الحدوث لذا كان من الضرورى التنقيب عن اهداف حيويه جديدة داخل الطفيل قابله للاستهداف بواسطه عقاقير اخرى.

**الطرق المستخدمه و النتائج**

اوضحت الدراسه باستخدام تقنيه إعاقه تكوين الحمض النووى الريبوزى

(RNA interference)ان انزيم(Thioredoxin Glutathione Reductase, TGR)

المحتوى على عنصر السيلينيوم هو إنزيم ضروري وفريد لحياة طفيل البلهارسيا، فعند اعاقة تكوين هذا الانزيم داخل ديدان البلهارسيا وجدنا تعرض البلهارسيا للموت في خلال اربعة ايام. و قد اوضحت الدراسة ايضا ان عقار الايرانوفين (auranofin) هو عقار فعال في تثبيط عمل هذا الانزيم كليا (Ki = 10 nM) و قتل طفيل البلهارسيا في مزرعة خارجية عند معدلات فسيولوجية .(Ki = 5 nM) إضافة الى ذلك فقد اكدت هذه الدراسة ان هذا العقار يتسبب في انخفاض ديدان البلهارسيا بمعدل 60% في فئران التجارب المصابة بهذا المرض. هذا وقد القت الدراسة الضوء على اثنين من العقاقير المستخدمة سالفا لعلاج البلهارسيا على انهما عقاقير مثبطة لعمل هذا الانزيم مما يؤكد الدور المحوري لهذا الانزيم و امكانيه استخدامه كهدف حيوي جديد لعلاج البلهارسيا.

ا**لخلاصه**

ففي المجمل العام،اكدت نتائج الدراسة الحالية ان إنزيم (TGR) في طفيل البلهارسيا هو هدف حيوي مثالي يمكن إستهدافه بواسطة العقاقير ومن ثم يمكن القضاء على البلهارسيا بالطرق الكيميائية.

والجدير بالذكر ان هذة الدراسة هي الاولي من نوعها في إثبات مثالية هدف حيوي في طفيل البلهارسيا باستخدام كلا من التقنية الجينية والبيوكيميائية علي السواء.
