# Supplementary material for: Thioredoxin Glutathione Reductase from Schistosoma mansoni: An Essential Parasite Enzyme and a Key Drug Target
Source: PLoS Med. 2007 Jun 19;4(6):e206. doi: 10.1371/journal.pmed.0040206 (PMC1892040; doi:10.1371/journal.pmed.0040206)
Supplement: Supplementary file 4 [file pmed.0040206.sd002.doc]

ABSTRACT

Background

Schistosomiasis is a widespread and devastating tropical disease affecting more than 200 million people. No vaccine is available and praziquantel the

only drug extensively utilized is currently administered more than 100 million people yearly. Because praziquantel resistance may develop it is essential

to identify novel drug targets.

Methods and Findings

Using RNA interference we found that the unique, selenium-containing parasite enzyme thioredoxin glutathione reductase (TGR) is essential for parasite

survival; after silencing TGR expression parasite death occurred within four days. We also found that auranofin was identified as an efficient inhibitor

of pure TGR (Ki = 10 nM), able to rapidly kill parasites in culture at physiological levels (5 µM), and to provide partial cures of infected mice (worm

burden reductions of ~60%). Furthermore, two previously used antischistosomal compounds were efficient TGR inhibitors, suggesting that TGR was a key

target during therapy with those compounds.

Conclusions

Collectively, our results indicate that parasite TGR meets all the major criteria to be a key target for anti-schistosomal chemotherapy. This is the

first validation of a Schistosoma drug target using a convergence of both genetic and biochemical approaches.

Résumé

Contexte:

La Schistosomiase est une maladie tropicale répandue et dévastatrice affectant plus de 200 millions de personnes. Aucun vaccin n’est disponible et le praziquantel est le seul médicament efficace actuellement administré à plus de 100 millions de personnes chaque année. En raison des risques de résistance au praziquantel, il est essentiel d'identifier de nouvelles cibles médicamenteuses.

Méthodes et Résultats :

En utilisant des expériences d'interférence à l'ARN, nous avons montré qu'une enzyme séléniée unique au parasite, la thioredoxine glutathion réductase (TGR), est essentielle à la survie parasitaire ; après extinction de l’expression de la TGR la mort parasitaire est effective dans les 4 jours. Nous avons également démontré que l'auranofine (AF) est un excellent inhibiteur de la TGR pure isolée (K_i_ = 10 nM), capable de tuer rapidement les parasites en culture à des doses physiologiques (5 µM), et de conduire à une guérison partielle des souris infectées (réduction de la charge en vers d’environ 60%). De plus, deux composés connus pour leurs propriétés schistosomicides sont des inhibiteurs puissants de la TGR, suggérant que la TGR est une enzyme clef lors du traitement avec ces composés.

Conclusions

Pris ensemble, nos résultats indiquent que la TGR du parasite présente tous les critères importants pour être considérée comme une cible majeure d’une chimiothérapie antischistosome. C’est la première validation d'une cible moléculaire spécifique du *Schistosoma* combinant une approche à la fois génétique et biochimique.
