# Supplementary material for: Correction: Thioredoxin Glutathione Reductase from Schistosoma mansoni: An Essential Parasite Enzyme and a Key Drug Target
Source: PLoS Med. 2007 Aug 28;4(8):e264. doi: 10.1371/journal.pmed.0040264 (PMC1952206; doi:10.1371/journal.pmed.0040264)
Supplement: Figure S1 — (407 KB DOC) [file pmed.0040264.sg001.doc]

Supplemental Fig. 1. Chemical structures of compounds used in this study as inhibitors of thioredoxin-glutathione

reductase (TGR) of *Schistosoma mansoni*.
